# Motion blur invariant for estimating motion parameters of medical ultrasound images

**DOI:** 10.1038/s41598-021-93636-4

**Published:** 2021-07-12

**Authors:** Barmak Honarvar Shakibaei Asli, Yifan Zhao, John Ahmet Erkoyuncu

**Affiliations:** 1 Centre for Life-Cycle Engineering and Management, School of Aerospace, Transport and Manufacturing, Cranfield University, Cranfield, Bedfordshire MK43 0AL UK; 2 Czech Academy of Sciences, Institute of Information Theory and Automation, Pod vodárenskou věží 4, 18208 Prague 8, Czech Republic

**Keywords:** Mathematics and computing, Engineering, Electrical and electronic engineering

## Abstract

High-quality medical ultrasound imaging is definitely concerning motion blur, while medical image analysis requires motionless and accurate data acquired by sonographers. The main idea of this paper is to establish some motion blur invariant in both frequency and moment domain to estimate the motion parameters of ultrasound images. We propose a discrete model of point spread function of motion blur convolution based on the Dirac delta function to simplify the analysis of motion invariant in frequency and moment domain. This model paves the way for estimating the motion angle and length in terms of the proposed invariant features. In this research, the performance of the proposed schemes is compared with other state-of-the-art existing methods of image deblurring. The experimental study performs using fetal phantom images and clinical fetal ultrasound images as well as breast scans. Moreover, to validate the accuracy of the proposed experimental framework, we apply two image quality assessment methods as no-reference and full-reference to show the robustness of the proposed algorithms compared to the well-known approaches.

## Introduction

Ultrasound is a sound wave with a frequency that exceeds 20 kHz above the audible range of humans. When ultrasound waves interact with the tissues due to the biophysics nature, it tends to obey the law of geometric optics. As they strike with the tissues, they interact by means of reflection, refraction, scattering and attenuation. Some of these interactions can reduce the intensity of the ultrasound waves in the form of noise. In any imaging system, when there exists an undesirable source of information, it will tamper with the size of the objects in the image, blur the edges or deteriorate the details of the image and is often termed as noise. Based on the mathematical model there are two types of noise such as multiplicative and additive noise. Speckle is one such signal dependent noise which hampers the quality of the imaging modality and has the characteristic feature of multiplicative noise. They occur in almost all types of coherent imaging systems such as acoustic, laser and synthetic aperture radar imagery (SAR). This type of noise exists when the sound waves of the same size or scale interacts to the sound wavelength that in turn effects the image resolution and contrast, causing the ultrasound image to be degraded. Improving the quality of ultrasound images is a difficult task because their characteristic feature is speckle. Speckle arises from signal interference caused by tissue microinhomogeneities (tissue cells, capillaries, blood cells, etc). This coherent summation of back scattered signals forms a spatial distribution of speckle that is specific to the density and distribution of the scatterers and thus to the nature of the tissue^1,2^. Image blurring is yet another distortion in the ultrasound images. Blurring acts as a low pass filter and attenuates higher spatial frequencies. Most of the blurred images can be approached by the convolution integrals^3^. The blurring in medical images causes a definite limit on the amount of detail that can be visualized where in it spreads the image of small objects into surrounding background area, characterized by a Point-Spread Function (PSF) or impulse response. The PSF is the output of the imaging system for a single point object. Thus, the blurring processes considered are linear and they are spatially invariant PSF. Therefore, to extract the information which is hidden in the blurred images the artefacts must be removed at the pre-processing phase. The analysis of blurred images is often carried out by first deblurring the images and then applying standard methods for further analysis^4^. The features in this paper are thus invariant to centrally symmetric blur.

**Figure 1:**
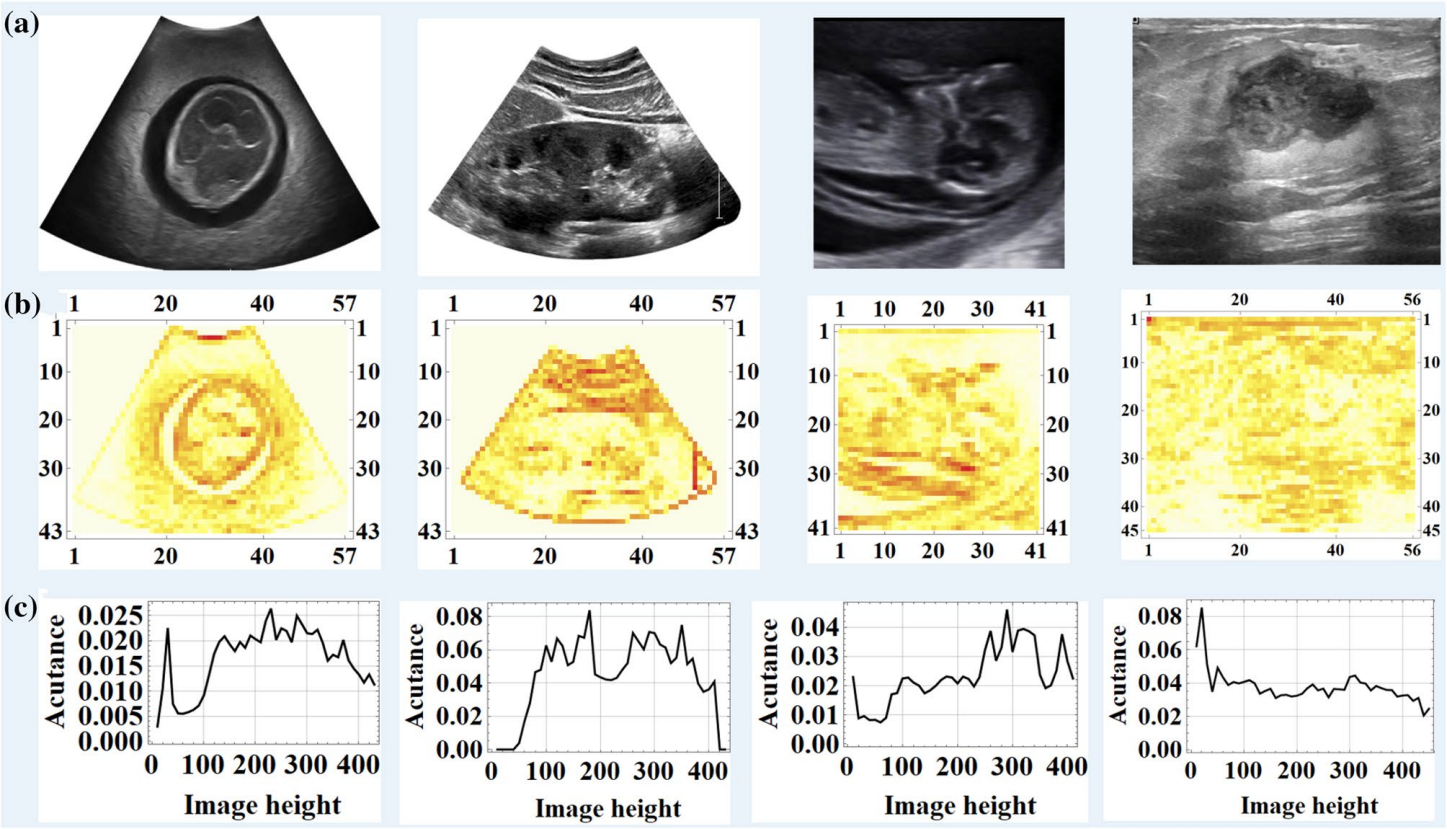
(**a**) Various ultrasound images, from left to right: Fetal phantom, kidney, third trimester fetus and breast cancer (malignant), (**b**) acutance map, (**c**) acutance distribution along the vertical axis.

In classical image processing, acutance can be considered as a characteristic of image based on its subjective evaluations which are influenced by the contrast along edges of image^5,6^. Whereas the observed sharpness is a combination of acutance and resolution, only one criterion can be tuned during image acquisition. Since there is a connection between acutance and sharpness of an image, based on judgement’s of people, the higher acutance results in a sharper image, which may not guarantee higher resolution. Figure 1 shows four different ultrasound images including different level of observed blur. The second row of the figure illustrates the acutance map derived from acutance calculation by dividing the image into 10 blocks. Blocks with higher acutance are sharper. Furthermore, the last column represents the calculation of the acutance distribution along the vertical axis.

Blur is a very common problem in image analysis that causes crisis in imaging system identification/recognition^7,8^. Object movement, focusing on a wrong area, sensor’s sensitivity to light and lens calibration are possible sources to develop blur issue in acquired images. There are various types of blur, but two of them are very common: motion blur and defocus blur. Motion blur is a result of altered position of a moving object while the defocus blur is due to an imprecise focal length alignment during image acquisition. A precise estimation of PSF can be obtained based on the image blur type and blur information which is called blur kernel (blur mask) and can be used for recovering of the original image from its distorted version. Some filters such as Gaussian filter can significantly suppress speckles and enhance contrast, but these algorithms cause blurry artefacts at edges and borders of image. There are several advanced approaches to solve this concern about the effect of blur^9–11^. As ultrasound data are prone to blur and speckle, classical image algorithms may generate lossy information of the deblurred image from an original degraded image based on its boundaries and edges. The level of blurriness which is convolved with a sharp image could be measured using PSF in an ultrasonic imaging system. In other words, the PSF with different kernel types represents how information is extracted from the object during the image acquisition. One the most common PSF kernel is the Gaussian distribution which has a wide application in imaging technologies^12^. There are many transformational and geometrical degradation on image processing in real applications that the imaging systems still operate on its normal mode in these non-ideal circumstances. Many literature reported different case studies in the area of pattern recognition by using the invariant characteristics of images^13–15^. Despite classical image processing applications in object recognition invariants, there are very limited research studies in medical imaging about blur model and image deblurring as an inverse problem. In the presence of noise *n*(*x*, *y*), we can model the degraded version, *g*(*x*, *y*), of a clean image *f*(*x*, *y*) by using the 2-D convolution (denoted by $*$) as follows:

1 $$\begin{aligned} g(x,y)=f(x,y) *h(x,y) + n(x,y), \end{aligned}$$

where *h*(*x*, *y*) is the PSF of the blurry system and (*x*, *y*) represents a 2-D spatial pixel coordinate of image plane. The PSF represents blur while other degradations are captured by the noise term *n*(*x*, *y*). This blurring effect causes a significant reduction in the sharpness of compound images, especially in ultrasound motion sequences^16^. Generally, the more frames used for compounding, the greater the improvement in image quality and the greater potential for motion blurring. This results in a trade-off between improving image quality and minimizing motion blurring. Invariant features play an important role in pattern recognition and shape detection as image characteristics may stand consistent under particular transformations such as scaling, translation and rotation^17,18^. Along with some research targeting to estimate the PSF, noise and speckle parameters (see^19^ for example), there are several successful approaches using the theory of image invariant analysis for ultrasound image segmentation and classification (see^20^ and^21^ for a survey). Although removing noise and speckle have been frequently been studied, motion blur investigation requires a wide range of estimation of the PSF parameters and evaluating the recovered sharp image. Especially for ultrasound images, this could be experienced in tissue edges or boundaries. Unlike the blur performance in classical photography, in ultrasound imaging system, the motion length increases (the motion angle changes) due to the form of ultrasound beam which is related to its distance from the ultrasound transducer. Since the shape of probes are mostly circular, the direction of the radiated ultrasound beams could be conducted to the center of transducer according to its geometry^22^. However, the radiated beams have a significant effect on the motion parameters including the blur length and angle that could be varied according to the position of probe during the scanning process. Levin *et al.* showed that a parabolic integration could lead to motion blur which is invariant to the speed of the movable object for omitting blur estimation^23^. They proposed a single deconvolution method to eliminate blur without calculation of the object velocity. Different from their work, the authors in^23,24^ developed a prototype camera including a sensor, two motion stages and their controllers to show the concept of deblurring using custom hardware. Both studies acknowledged the image stabilization hardware as a proper motion invariant performance at the time of the image acquisition.

One of the robust image analysis tools that plays an important role in the image filtering area is the Fourier transform. This powerful approach uses a complex-exponential kernel to decompose an image into its constituent sine waves with particular frequencies and amplitudes. The Fourier transform operates as a system that its input is the spatial domain representation of image and the output describes the frequency domain of the same image using spectrum features which are amplitude and phase. One robust application of Fourier transform in image analysis is Wiener filter which is an optimal stationary linear filter for images degraded by blur mask and additive noise. The Wiener process requires the assumption that the spectrum features of image and noise are known. This process can be considered as a blind deconvolution that would estimate the level of blur by calculating the PSF and reconstructing the original image which is already mapped into the frequency domain for performing deconvolution.

In this study, we first focus on developing a novel algorithm to investigate the motion blur invariants based on spectrum analysis of a linear motion PSF. It will be done by considering the imaging system in the presence and absence of noise. Second, the derivation of the blur invariant will be formulated in the geometric moment domain in the absence of noise. Moreover, we estimate the motion blur parameters including blur angle and blur length for ultrasound images based on the obtained frequency and moment domain invariants. This algorithm can be recognized as a blind deconvolution to find the motion blur PSF and the original sharp image using its degraded version. In addition to the above discussion, we report a comparative study of the proposed blind deconvolution achieved by frequency domain analysis with the existing algorithms such as the cepstrum approach, blind restoration method, and equivalent Gaussian representation. The experimental results show that the proposed invariant frequency domain algorithm is robust to the variation in the PSF parameters estimation for blur length and angle. Finally, the image quality metrics (either a full-reference method that evaluates a test image with respect to a reference image to quantify their visual similarity or a no-reference method that calculates the no-reference image quality score for an image using the Blind/Referenceless Image) has been used for validating the proposed method in comparison with the existing approaches. These scores indicate that the new algorithm performs better image quality than the other methods.

## Motion blur formulation

Equation (1) represents the model of an imaging system including blur and noise by using 2D convolution of original image and PSF. Based on the invariant features of PSF, in most cases of imaging structures, the blur function is a spatially continuous process. In many pre-processing techniques such as image recognition and reconstruction, the image base is discrete, while the blur model could be considered in its continuous form and can be converted to a discrete convolution model. There are various types of motion blur which are generated during image acquisition based on the sensors, scene and exposure time of image capturing. In blur model formulation, it can be used translation, rotation and scaling of PSF or any possible combination of these transformations. According to the above description of motion blur, we can define the following continuous PSF in terms of the blur angle $\theta$ and blur length $L=v_0t$ (where $v_0$ is the velocity of camera movement and *t* is the exposure time) as:

2 $$\begin{aligned} h(x,y)=\frac{1}{L}\int _{-L/2}^{L/2} \delta (x-t\cos \theta )\delta (y-t\sin \theta ) \mathrm dt, \end{aligned}$$

where $\delta (.)$ is the Dirac function. The discrete form of (2) is not easily captured in a close form expression in general^25^. In case of linear horizontal motion, the blur angle is zero $(\theta =0^{\circ })$ and $h(x,y)=\frac{1}{L} \delta (y) \,\,\text {for} \,\, \left| x \right| \le L/2$. For linear vertical motion, the blur angle is $90^{\circ }$ and $h(x,y)=\frac{1}{L} \delta (x) \,\,\text {for} \,\,\left| y \right| \le L/2$. We use an approximated discrete form of (2) by applying the relation of discrete delta function to rectangle function, $\Pi (.)$, as follows:

3 $$\begin{aligned} h(n,m)&= \frac{1}{L}\sum _{k=-\infty }^{+\infty } \delta (n-k\cos \theta )\delta (m-k\sin \theta ) \nonumber \\&= \frac{1}{L}\Pi (m\cos \theta -n\sin \theta ). \end{aligned}$$

**Figure 2:**
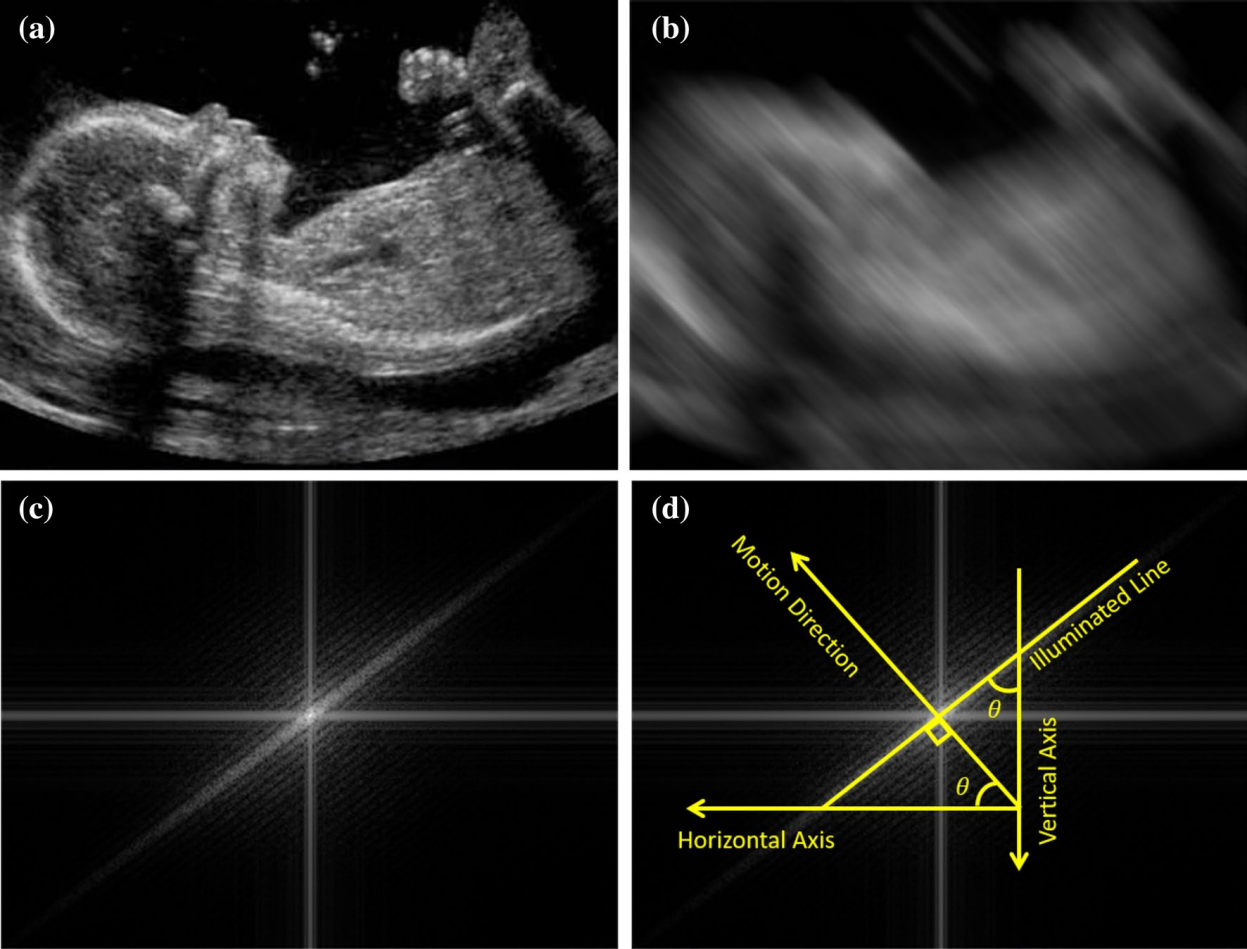
(**a**) Fetus ultrasound image, (**b**) the blurred version of image generated by linear motion, (**c**) Fourier transform of image (**b**), (**d**) the geometrical representation of blur angle $\theta$ and linear motion direction shown in (**c**).

It is possible to show that the behavior of the Eqs. (2) and (3) is almost the same. Figure 2a,b show a non-blur fetus image and its blurred version generated by a linear motion, respectively. By transforming the gradient of the blurred version of image into the frequency domain, a set of parallel arrays of illuminated lines could be distinguished as shown in Fig. 2c. The geometry of these arrays propagation is illustrated in Fig. 2d as a mark for PSF role in frequency domain. It can be seen from this figure that the motion blur angle $\theta$ could be defined as the angle between the illuminated lines and the vertical axis of image which is the same as the angle between the motion direction and the horizontal axis of image. Note that, the blur length (L) as the second parameter of motion blur is identical to the distance among the adjoining illuminated lines^26^. The estimation process of the blur parameters (length and angle) can be completed by calculating the geometrical orientation of these illuminated lines.

**Figure 3:**
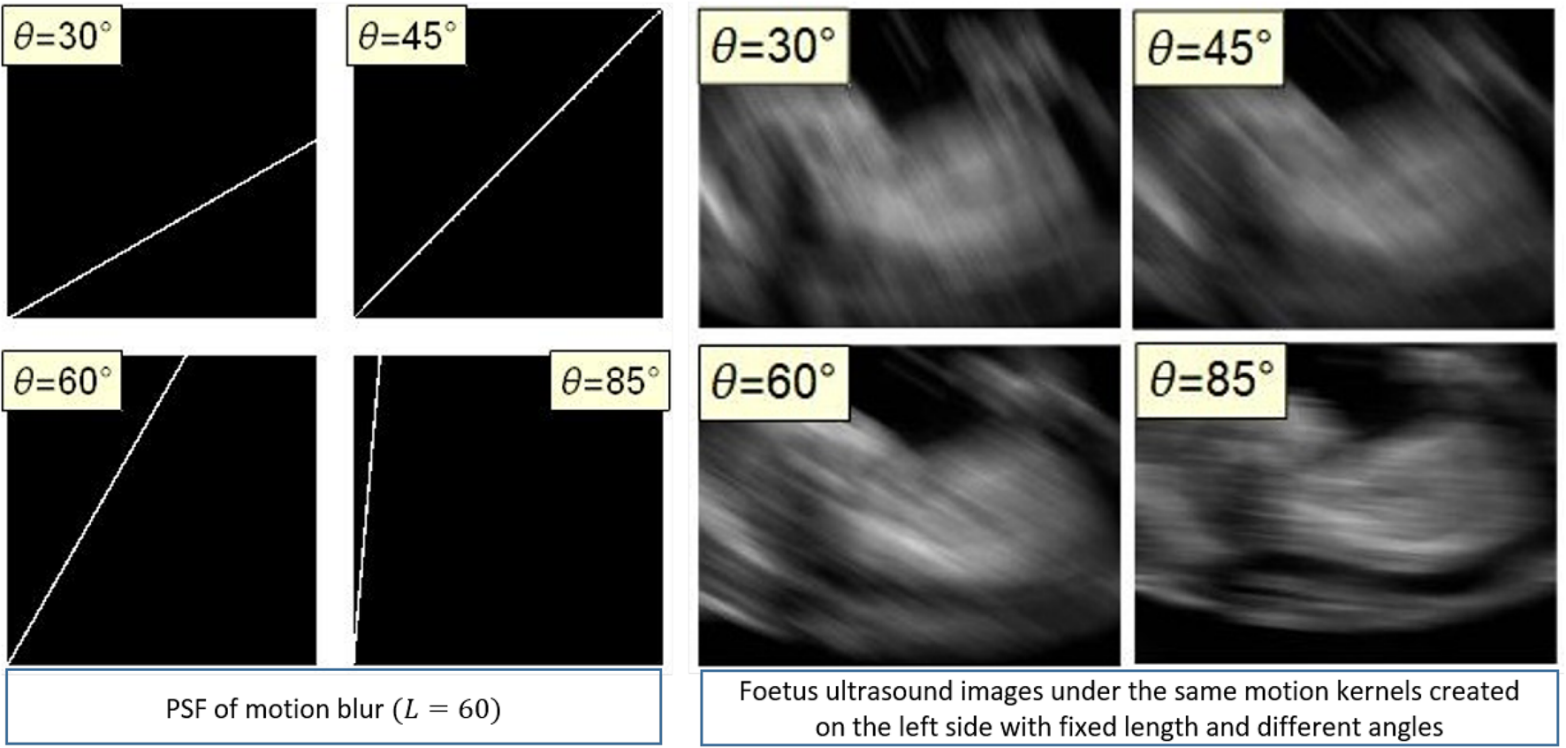
Understanding of motion blur. Left: PSF of motion blur in spatial domain for $L=60$ and different angles $(\theta =30^{\circ },45^{\circ },60^{\circ },85^{\circ })$ and right: Fetus ultrasound images with fixed length and different angles of motion.

Figure 3 shows the density graph of the discrete PSF for $L=60$ and different values of $\theta$ on the left side. On the right side of the same figure, the fetus ultrasound image shown in Fig. 2a is simulated with a fixed length of motion blur and the same various motion angles to illustrate the visualization of motion blur. As another representation of discrete motion PSF is the matrix description. The following matrices show the PSF for motion blur with $L=7$ and varying $\theta$ from left to right, respectively $(30^{\circ },45^{\circ },60^{\circ },85^{\circ })$.

$$\begin{aligned}&\left( \begin{array}{ccccccc} \frac{1}{7} &{} 0 &{} 0 &{} 0 &{} 0 &{} 0 &{} 0 \\ \frac{1}{7} &{} \frac{1}{7} &{} 0 &{} 0 &{} 0 &{} 0 &{} 0 \\ 0 &{} \frac{1}{7} &{} 0 &{} 0 &{} 0 &{} 0 &{} 0 \\ 0 &{} 0 &{} \frac{1}{7} &{} 0 &{} 0 &{} 0 &{} 0 \\ 0 &{} 0 &{} \frac{1}{7} &{} 0 &{} 0 &{} 0 &{} 0 \\ 0 &{} 0 &{} 0 &{} \frac{1}{7} &{} 0 &{} 0 &{} 0 \\ 0 &{} 0 &{} 0 &{} \frac{1}{7} &{} \frac{1}{7} &{} 0 &{} 0 \\ \end{array} \right) \,\,\left( \begin{array}{ccccccc} \frac{1}{7} &{} 0 &{} 0 &{} 0 &{} 0 &{} 0 &{} 0 \\ 0 &{} \frac{1}{7} &{} 0 &{} 0 &{} 0 &{} 0 &{} 0 \\ 0 &{} 0 &{} \frac{1}{7} &{} 0 &{} 0 &{} 0 &{} 0 \\ 0 &{} 0 &{} 0 &{} \frac{1}{7} &{} 0 &{} 0 &{} 0 \\ 0 &{} 0 &{} 0 &{} 0 &{} \frac{1}{7} &{} 0 &{} 0 \\ 0 &{} 0 &{} 0 &{} 0 &{} 0 &{} \frac{1}{7} &{} 0 \\ 0 &{} 0 &{} 0 &{} 0 &{} 0 &{} 0 &{} \frac{1}{7} \\ \end{array}\right) \\&\left( \begin{array}{ccccccc} \frac{1}{7} &{} 0 &{} 0 &{} 0 &{} 0 &{} 0 &{} 0 \\ 0 &{} \frac{1}{7} &{} \frac{1}{7} &{} 0 &{} 0 &{} 0 &{} 0 \\ 0 &{} 0 &{} 0 &{} \frac{1}{7} &{} \frac{1}{7} &{} 0 &{} 0 \\ 0 &{} 0 &{} 0 &{} 0 &{} 0 &{} \frac{1}{7} &{} \frac{1}{7} \\ 0 &{} 0 &{} 0 &{} 0 &{} 0 &{} 0 &{} \frac{1}{7} \\ 0 &{} 0 &{} 0 &{} 0 &{} 0 &{} 0 &{} 0 \\ 0 &{} 0 &{} 0 &{} 0 &{} 0 &{} 0 &{} 0 \\ \end{array} \right) \,\, \left( \begin{array}{ccccccc} \frac{1}{7} &{} \frac{1}{7} &{} \frac{1}{7} &{} \frac{1}{7} &{} \frac{1}{7} &{} \frac{1}{7} &{} 0 \\ 0 &{} 0 &{} 0 &{} 0 &{} 0 &{} 0 &{} \frac{1}{7} \\ 0 &{} 0 &{} 0 &{} 0 &{} 0 &{} 0 &{} 0 \\ 0 &{} 0 &{} 0 &{} 0 &{} 0 &{} 0 &{} 0 \\ 0 &{} 0 &{} 0 &{} 0 &{} 0 &{} 0 &{} 0 \\ 0 &{} 0 &{} 0 &{} 0 &{} 0 &{} 0 &{} 0 \\ 0 &{} 0 &{} 0 &{} 0 &{} 0 &{} 0 &{} 0 \\ \end{array} \right) \end{aligned}$$

## Motion blur invariant

In this section, we establish a new frequency and moment blur invariant based on a linear motion PSF for degraded images. Since an imaging system can be modeled as a 2D convolution in (1), it is possible to transform this equation to the Fourier or moment domains. For frequency analysis, we consider the imaging system in the presence and absence of noise, respectively. For moment domain analysis, we only derive the invariant properties of ultrasound images in the absence of noise.

### Frequency domain invariant

#### Effect of noise

In the presence of noise, the degradation model in (1) can be expressed in the Fourier domain as:

4 $$\begin{aligned} G(u,v)=F(u,v)H(u,v)+N(u,v), \end{aligned}$$

where *G*(*u*, *v*), *F*(*u*, *v*), *H*(*u*, *v*) and *N*(*u*, *v*) are the frequency responses of the observed image, original image, PSF, and noise, respectively. The Wiener deconvolution method has widespread use in image deconvolution applications, as the frequency spectrum of most visual images is fairly well behaved and may be estimated easily^27^. Here, the target is to find $\lambda (x,y)$ in the way that $\widehat{f}(x,y)$ can be approximated as a convolution, that is, $\lambda (x,y) *g(x,y)$, to minimize the mean square error, where $\widehat{f}(x,y)$ is an estimation of *f*(*x*, *y*). The Wiener deconvolution filter provides such a $\lambda (x,y)$. The filter is described in the frequency domain^28^:

5 $$\begin{aligned} \Lambda (u,v)=\frac{\overline{H}(u,v)S(u,v)}{\left| H(u,v)\right| ^2 S(u,v)+N(u,v)}, \end{aligned}$$

where *S*(*u*, *v*) the mean power spectral density $(S(u,v)=\mathbf{E} \{\left| F(u,v)\right| ^2\})$ of the original image, *f*(*x*, *y*) and the vinculum denotes complex conjugation. Using this technique to find the best reconstruction of a noisy image can be compared with other algorithms such as Gaussian filtering.

#### Absence of noise

If noise is neglected, Eq. (4) can be reduced to a simple product of *F*(*u*, *v*) and *H*(*u*, *v*). The Fourier transform of (2) can be written as the following *sinc* function:

6 $$\begin{aligned} H(u,v)={{\,\mathrm{sinc}\,}}\left( \frac{L\left( u\cos \theta + v\sin \theta \right) }{2 \pi }\right) . \end{aligned}$$

**Figure 4:**
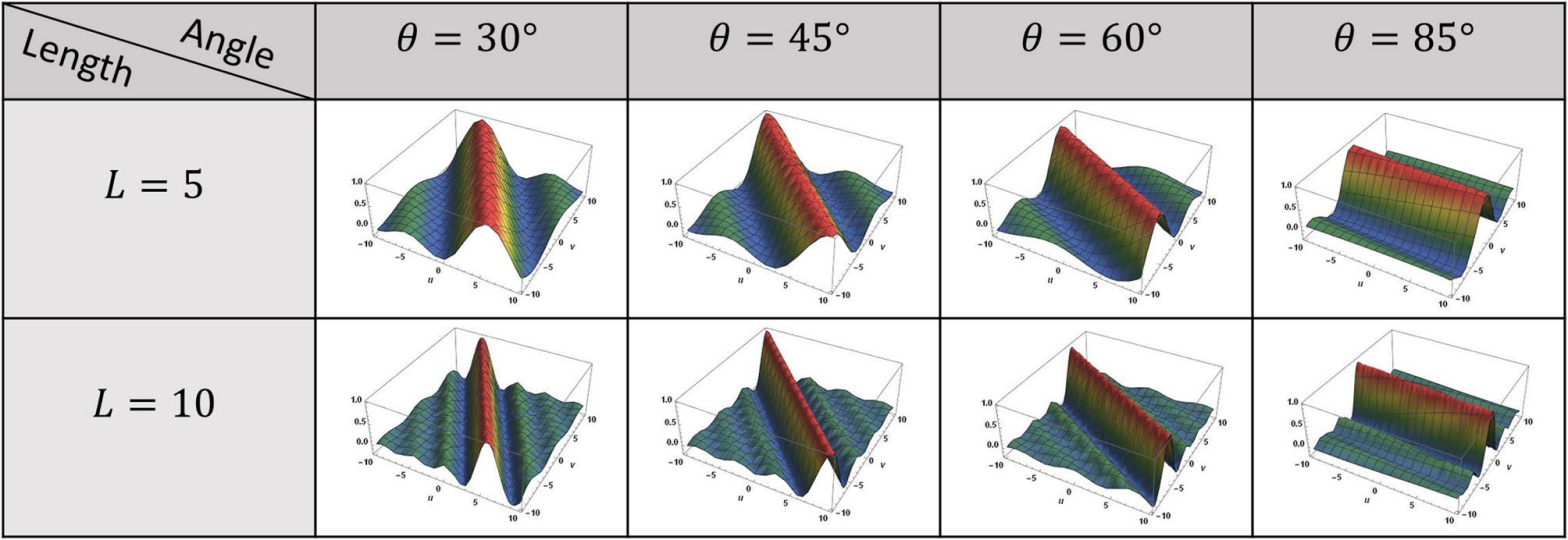
PSF of motion blur in the Fourier domain for different lengths $(L=5,10)$ and angles $(\theta =30^{\circ },45^{\circ },60^{\circ },85^{\circ })$.

Figure 4 shows the Fourier transform of the PSF of motion blur with different values of blur lengths and angles. The figure illustrates that the blur is effectively a low-pass filtering operation and has spectral zeros along characteristic lines.

The imperfections in the image formation process are modeled as passive operations on the data, i.e., no “energy” is absorbed or generated. Consequently, for spatially continuous blurs the PSF is constrained to satisfy the global energy preserving $\int _{-\infty }^{\infty }\int _{-\infty }^{\infty } h(x,y)dxdy=1$ which also supports the spectral condition of $H(0,0)=1$.

By using the reduced version of (4) in the absence of noise, we get

7 $$\begin{aligned} G(u,v)=F(u,v){{\,\mathrm{sinc}\,}}\left( \frac{L\left( u\cos \theta + v\sin \theta \right) }{2 \pi }\right) . \end{aligned}$$

To find the motion blur parameters (length and angle), we set both frequencies (*u*, *v*) to (0, 1) and (1, 0), respectively. By defining $a={{\,\mathrm{sinc}\,}}^{-1} \left[ G(0,1)/F(0,1)\right]$ and $b={{\,\mathrm{sinc}\,}}^{-1} \left[ G(1,0)/F(1,0)\right]$, we derive $\theta =\tan ^{-1} (a/b)$ and $L=2\pi a \csc \theta$. Finally, by substituting the obtained angle and length of motion blur in terms of low frequencies, $(u,v)\in \{0,1\}$, in (7), we can derive a frequency invariant scheme for motion blur as:

8 $$\begin{aligned} \xi (u,v)=\frac{G(u,v)}{F(u,v)}={{\,\mathrm{sinc}\,}}\left[ a(u\cot \theta +v)\right] ={{\,\mathrm{sinc}\,}}(av+bu). \end{aligned}$$

It means that there is a relationship between the original and degraded images’ spectrum (*F*(*u*, *v*) and *G*(*u*, *v*)) with their low frequency components (*F*(0, 1), *F*(1, 0), *G*(0, 1) and *G*(1, 0)). Equation (8) shows the proposed blur invariant features in Fourier domain - called $\xi (u,v)$ - for all range of frequencies which is independent of the motion blur kernel parameters. In “Experiments” section, we show some of these invariants.

### Motion blur invariant in moment domain

As we discussed in the Introduction, the various image descriptors such as image moments and frequency spectrum are invariant to translation, scaling, rotation and convolution. Therefore, the computation of motion blur on ultrasound images could be achieved via image descriptors since the speckle size depends to the ultrasound beam diameter that is varying over the image. Here, we formulate a set of geometric moment invariant reported in^29,30^. In the absence of noise *n*(*x*, *y*) in (1), there is a relation between the degraded image moments and its clean version moments and PSF moments^31^ as:

9 $$\begin{aligned} m_{pq}^{(g)}=\sum _{k=0}^{p} \sum _{l=0}^{q} \left( {\begin{array}{c}p\\ k\end{array}}\right) \left( {\begin{array}{c}q\\ l\end{array}}\right) m_{kl}^{(h)} m_{p-k,q-l}^{(f)}, \end{aligned}$$

where $m_{pq}^{(g)}$, $m_{pq}^{(f)}$ and $m_{pq}^{(h)}$ are the two-dimensional $(p+q)^{\text {th}}$ order geometric moments of the observed image, original image, and PSF respectively. The two-dimensional $(p+q)^{\text {th}}$ order geometric moments of the original image is defined by

10 $$\begin{aligned} m_{pq}^{(f)}=\int _{R^2} f(x,y)x^p y^q \mathrm dx \mathrm dy. \end{aligned}$$

The geometric moments of the PSF can be calculated from the motion function (using sifting property^32^ of Dirac delta function) with Eq. (2):

11 $$\begin{aligned} m_{pq}^{(h)}={\left\{ \begin{array}{ll} \frac{\left( L/2 \right) ^{p+q} \left( \cos \theta \right) ^p \left( \sin \theta \right) ^q}{p+q+1},&{} p+q=\text {even}\\ 0, &{} p+q=\text {odd.} \end{array}\right. } \end{aligned}$$

Substituting (11) in (9) and expanding the observed image moments in terms of the original image moments, it is clear that the zeroth and first invariant moments could be found directly $(m_{00}^{(g)}=m_{00}^{(f)} \,,\,m_{01}^{(g)}=m_{01}^{(f)}\,,\,m_{10}^{(g)}=m_{10}^{(f)})$. The second orders invariant can be obtained as follows:

12 $$\begin{aligned} m_{11}^{(g)}&=(L^2/24) \sin 2\theta \,\, m_{00}^{(f)}+m_{11}^{(f)}\nonumber \\ m_{20}^{(g)}&=(L^2/12) \cos ^2 \theta \,\, m_{00}^{(f)}+m_{20}^{(f)}\nonumber \\ m_{02}^{(g)}&=(L^2/12) \sin ^2 \theta \,\, m_{00}^{(f)}+m_{02}^{(f)} \end{aligned}$$

From the last two equations in (12), we can find the direction of motion blur in terms of the second order moments as follows:

13 $$\begin{aligned} \theta =\tan ^{-1} \left( \frac{m_{02}^{(g)}-m_{02}^{(f)}}{m_{20}^{(g)}-m_{20}^{(f)}} \right) ^{1/2}. \end{aligned}$$

Finally, by substituting (13) in the second equation of (12), the length of the motion blur could be obtained as:

14 $$\begin{aligned} L=2\sqrt{3} \left( \frac{m_{20}^{(g)}+m_{02}^{(g)}-m_{20}^{(f)}-m_{02}^{(f)}}{m_{00}^{(f)}}\right) ^{1/2}. \end{aligned}$$

Equations (9), (13) and (14) show that the moment invariants are a linear combination of their original moments, thus they maintain the capacity for feature analysis. Moreover, these derivations confirm that our proposed invariant scheme is matched with the ordinary moment invariants with respect to blur (convolution). In the result section, we evaluate these invariants of different orders ($\mathbb {M}_{(p+q)}$) for degraded ultrasound images by motion blur.

### Motion blur parameter estimation in ultrasound images

Based on the main principles of the image blur, PSF could be estimated in terms of two parameters of the motion blur: the angle and the length of motion, whose values are often non-existent by reason of the intrinsic nature of ultrasound motions and speckle. There are various solutions for blur parameters estimation but the well-known method is the blind image deblurring algorithms that evaluates the estimated parameters of the PSF as motion angle/length to obtain a sharp reconstructed version of degraded image^33,34^.

#### Estimating motion angle

As discussed earlier, the measurement of the parallel illuminated lines in Fourier domain is a way to estimate the motion angle. To get a high accuracy estimation for motion parameters, a piecewise-linear bilateral filter is applied to the proposed method^35^. Using the classical edge detection techniques, we are able to determine the edges on both sides of the middle illuminated line of the transformed PSF on Fourier domain. The derived edges can be split into different overlaid regions and the angles among each region and vertical axis will be measured based on motion direction. Therefore, this algorithm could be considered as an effective method for motion angle estimation.

#### Estimating motion length

By using the aforementioned method to determine the estimated value of motion angle, it is possible to take the discrete Fourier transform (DFT) of the motion blur PSF and it would be a discrete version of (6) as

15 $$\begin{aligned} H(u,v)={{\,\mathrm{sinc}\,}}\left[ \frac{L}{2 \pi } \left( \frac{u\cos \theta }{M}+\frac{v\sin \theta }{N}\right) \right] , \end{aligned}$$

where *M* and *N* are the width and height of image. Assuming $\omega =(u\cos \theta ) /M+(u\sin \theta )/N$, the solution of $H(\omega ) = 0$, which is the position of illuminated lines would develop the following formula:

16 $$\begin{aligned} \frac{u\cos \theta }{M}+\frac{v\sin \theta }{N}=\pm \frac{2k\pi }{L}. \,\,\,\, ; \,\,\,\, k=1,2,... \end{aligned}$$

Notice that when the image is square $(M=N)$, the above formula can be reduced to $u\cos \theta +v\sin \theta =\pm 2kN\pi /L$. Equation (16) shows the location of illuminated lines determined by *L* in spectrum analysis. It means that the blur length can be described as *d*, the distance between the image size $(M \times N)$ and two successive zeros of $H(\omega ) = 0$ as $L=(M \times N)/d$.

The pseudocode implementation of (5), (15) and (16) is given in Algorithm 1.

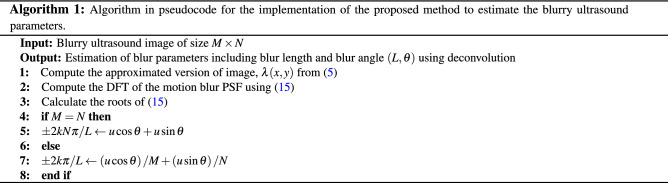



## Experiments

Three numerical experiments were conducted in order to prove the validity and the efficiency of the proposed methods. The first and the third experiments have been performed using two sets of videos (five slow and five fast scans) without any visual feedback in a trajectory (axially from head to toe or toe to head followed by moving the probe in the opposite direction after placing it in a perpendicular orientation) based on scans of a fetal phantom (SPACEFAN-ST, Kyoto Kagaku) by a convex transducer probe with a Telemed MicrUs Scanner (Telemed Ultrasound Medical Systems, Lithuania). In these experiments, different fetal scans (normal and anomaly), fetal spinal and fetal hydronephrosis scans have been used too. These scans were performed in a trajectory (axially from head to toe or toe to head followed by sagittally in the opposite direction) in a display-less mode. All images were extracted from different sets of videos. Since data are blurry, we have decided to obtain and present such data for the proposed frequency invariants algorithm. Moreover, in the second part of the third experiment, a malignant type of the breast cancer ultrasound image has been used^36^. In the second experiment, we used an 11 weeks’ gestation fetal ultrasound image. All examinations were performed in accordance with relevant guidelines and regulations. The local ethical review committee (the Urmia University of Medical Sciences) approved this study with its all experimental protocols. All dataset was obtained from pregnant women in the age range between 27 to 43 years old. Informed consent was obtained from all the participants. There were no subjects under 18 which needed parent/legal guardian.

### First experiment

Tables 1 and 2 show the slow and fast scans of the phantom and normal/anomaly fetal images with different motion blur levels. The blur invariants shown in (8) are denoted as $\xi (u,v)$ where the frequencies, *u* and *v*, are varied in random ranges. Each row of these tables illustrate the frequency invariant of slow/fast scans by computing the amplitude and phase parts of $\xi (u,v)$ for selected frequencies. It can be observed that the respective values of $\left| \xi (u,v)\right|$ and  change slightly for slow/fast captured motion blur. Since the results for values of the amplitude and phase of blur invariant are close enough for fast/slow scans, the proposed method is robust.

**Table 1:**
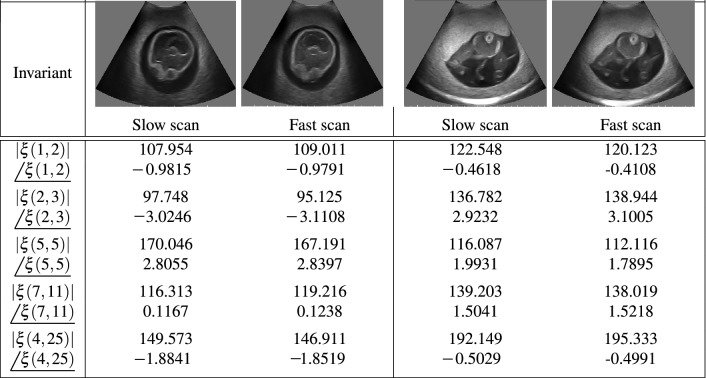
The values of the frequency invariants including amplitude $\left( \left| \xi (u,v)\right| \right)$ and phase () in radian with different values of (*u*, *v*) for slow and fast scan phantom ultrasound images followed by motion model.

**Table 2:**
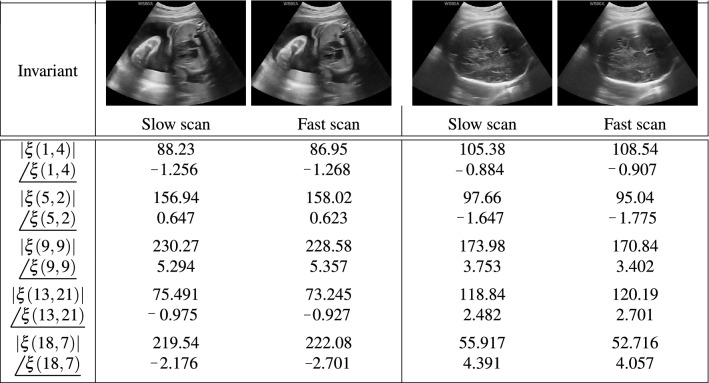
The values of the frequency invariants including amplitude $\left( \left| \xi (u,v)\right| \right)$ and phase () in radian with different values of (*u*, *v*) for slow and fast scan clinical real ultrasound images followed by motion model.

### Second experiment

An original 11 weeks’ gestation fetal ultrasound image was blurred by four different motion blur of the direction $30^{\circ }$, $45^{\circ }$, $60^{\circ }$ and $85^{\circ }$. The length of blurs for the corresponding directions were $L=20$, $L=40$, $L=30$ and $L=50$, respectively. For these five images we calculated blur moment invariants based on (9), (13) and (14) from zeroth order to fourth order (see Table 3). One can see from Table 3 how important it is to understand the theoretical properties of the moment invariants under various levels of motion blur. As we discussed in “Motion blur invariant in moment domain” section, the zeroth and the first invariant moments are the same (row $\mathbb {M}_{0}$ and $\mathbb {M}_{1}$ in table). On the other hand, all $\mathbb {M}_{2}$, $\mathbb {M}_{3}$ and $\mathbb {M}_{4}$ invariants provide a perfect stability. The advantage of the proposed geometric moment invariant scheme is its robustness in terms of the motion blur parameters’ variations. Despite the amount of blur length varies from 20 to 50 and the level of blur angle is varying from $30^{\circ }$ to $85^{\circ }$, there are very stable results for moment invariant features.

**Table 3:**
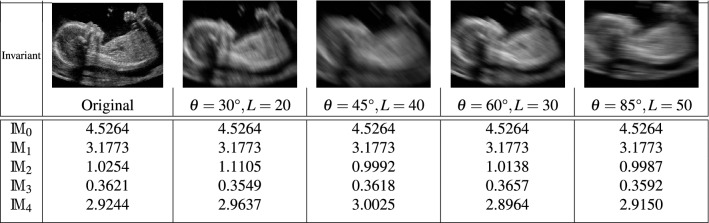
The values of the geometric moment invariants with different motion blur parameters of angle/length. $\mathbb {M}_{r}$ shows the invariant value of the moment order *r*, as discussed in “Motion blur invariant in moment domain” section.

### Third experiment

The last experiment is summarized in two tables. In the first Table 4, three sets of fetal phantom images are used to estimate motion blur parameters based on “Motion blur parameter estimation in ultrasound images” section. The table shows the setup, where the blurred frames are restored using three different deblurring algorithms as well as the proposed frequency domain method. Here, the first column shows the acquired phantom images denoted by single fast scan and two slow scans. The second column shows the images restored in cepstrum domain using algorithm in^37^. The method is based on noise-robust 2-D phase unwrapping and a noise-robust procedure to estimate the pulse in the complex cepstrum domain and initially applied for 2-D blind homomorphic deconvolution of medical B-scan ultrasound images. The third column illustrates the image restoration based on the spectrum of the blurred images and is supported on a weak assumption, which is valid for the most natural images and uses some modifications to the radon transform^38^. The last used algorithm for our comparison is shown in the fourth column. This method decouples motion and defocus blurs using equivalent Gaussian representation which leads to accurate estimation of underlying spatially varying defocus and motion blur PSFs from a single image^39^. Finally, the last column shows our proposed frequency domain motion blur estimation algorithm to recover blurry images. To evaluate the accuracy of the aforementioned algorithms compared with the proposed method, we use two criteria: (a) The blind/referenceless image spatial quality evaluator (BRISQUE) is applied to get a score for image measurement from a natural image model^40–42^. For this score, a lower value indicates a better subjective quality, (b) The structural similarity index (SSIM) is another perceptual metric that quantifies image quality degradation caused by noise or blur which are taken into account for this experiment. Higher SSIM matches with a better image reconstruction^42^. It can be observed that there are significant noticeable artefacts at the borders of the restored image using methods^37–39^. The proposed method performs competitively when compared to the existing methods. As indicated in the last column of the table for image quality scores, the bolded values represent the better restored images. It is worth noting that all methods are quite accurate in terms of blur angle and length estimation while the proposed method provides better image quality with respect to image scores, BRISQUE and SSIM. Table 5 presents the same deblurring processes using the clinical ultrasound images. The fetal spinal, fetal hydronephrosis and malignant breast cancer scans are shown in the first, second and third rows, respectively. The proposed approach represents better quality of the reconstructed images in terms of the introduced image quality criteria.

**Table 4:**
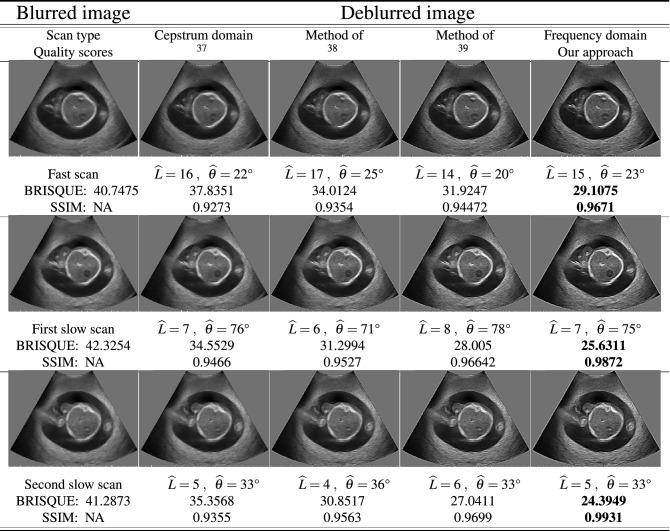
Fetal phantom image deblurring using three different methods compared with frequency domain (proposed scheme using estimated motion algorithm) and their corresponding BRISQUE/SSIM scores.

**Table 5:**
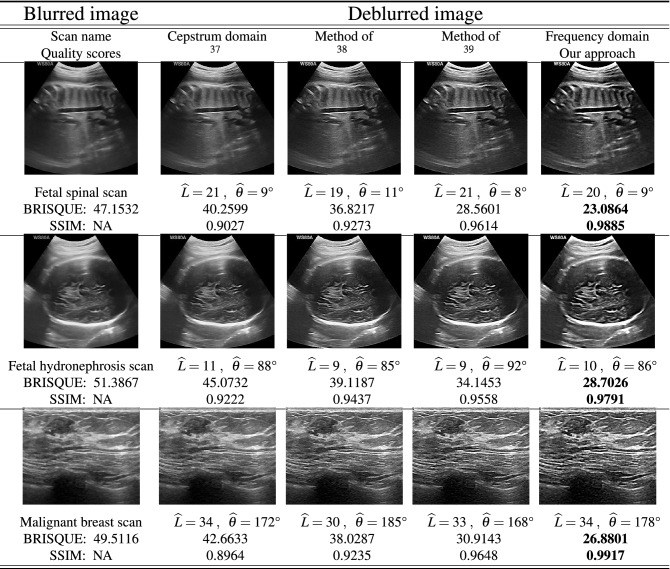
Fetal spinal, fetal hydronephrosis and Malignant breast image deblurring using three different methods compared with frequency domain (proposed scheme using estimated motion algorithm) and their corresponding BRISQUE/SSIM scores.

## Conclusion

In this paper, we have proposed a suitable model of fetal ultrasound imaging systems with respect to their motion blur phenomenon. The idea of invariant features of fast and slow scan of ultrasound images is developed in both frequency and moment domains. We studied the PSF behaviour of blurry ultrasound images in time-domain, moment domain, matrix form and frequency domain. An estimation algorithm of PSF in terms of blur angle and blur length is also proposed. Using the obtained PSF information, restoration of the motion blurred ultrasound images is performed in the frequency domain. Experiments demonstrate that better perceptual quality was obtained in the frequency domain. A comparative analysis of deblurring results obtained using the cepstrum and frequency domains are conducted using BRISQUE and SSIM scores. It has been observed that using frequency domain results are robust to the variation in the estimated PSF parameters.

## Data Availability

The data that support the findings of this study are available on request from the corresponding author, B. Honarvar. The data are not publicly available due to containing information that could compromise the privacy of research participants.
